# Trust-Wide Quality Improvement Project on Improving the Competence and Confidence of First On-call Doctors in Nottinghamshire Healthcare NHS Foundation Trust (NHCFT)

**DOI:** 10.1192/bjo.2025.10411

**Published:** 2025-06-20

**Authors:** Sowmy Murickal, Jonathan Wright, Joshua Bachra, Julie Rastall, Emma Poynton-Smith

**Affiliations:** Nottinghamshire Healthcare NHS Foundation Trust, Nottingham, United Kingdom

## Abstract

**Aims:** This project emerged in response to surveys conducted in 2022–23, which revealed first on-call doctors at NHCFT, perceived they were required to operate beyond their competency levels. Recognising this could negatively affect both patient safety, and training experience of resident doctors, we sought to improve their confidence and competence through structured support, education, and resource development.

The project’s aim was to ensure that no first on-call doctor would feel that they were working beyond their competency.

**Methods:** Cycle-1: The project began with anonymous baseline surveys using Hewson Confidence Tool, grade-specific focus groups which revealed a lack of knowledge in both clinical and practical aspects, increased stress due to untriaged workloads, and feelings of insufficient support from senior staff which contributed to widespread sense of being overwhelmed and impacted confidence and competence. Primary intervention included targeted on-call teaching sessions focussing on areas such as the role of on-call resident doctors, management of common tasks, seclusion reviews, legal frameworks, and escalation pathways.

Cycle-2: Observing the positive impact on doctors’ self-reported competency and confidence levels in the first PDSA cycle, Cycle 2 began with stakeholder engagement through listening events with first on-call doctors. We held discussions with key leaders, Director of Medical Education, Associate Director of Nursing, Deputy Associate Director of Physical Healthcare, ECG Trainer, Physical Healthcare Nurse, and Ward managers.

Subsequently, we designed and delivered a bespoke ‘First On-Call Workshop for Resident Doctors', held as two 90-minute sessions in September 2024. The workshops used interactive tools, case-based scenarios, and audio-visual aids on psychiatric medication side effects, managing psychiatric emergencies, risk assessment, escalation pathways, legal procedures e.g., Section 5(2), demonstration videos for ECG and catheterization, along with site orientation videos.

**Results:** Cycle-1: Results displayed a 7% increase in confidence and competence.

Cycle-2: Outcome measures displayed positive qualitative feedback and an increase in confidence in quantitative feedback by 17% in handling common on-call tasks and clinical scenarios.

For sustainability, online resources and tools, i.e., workshop materials, videos, and podcasts, were made accessible and included in Resident Doctors’ Survival Guide. Senior medics are now equipped with resources to facilitate these workshops, ensuring the project’s longevity.

**Conclusion:** This project contributed to enhancing the competence and confidence of first on-call doctors, thereby improving patient safety, and fostering a supportive learning environment within NHCFT. This initiative has underscored the importance of structured educational interventions and collaborative support systems in promoting both trainee and patient well-being.

